# Risk factors for agitation in home-cared older adults with dementia: evidence from 640 elders in East China

**DOI:** 10.3389/fnins.2023.1189590

**Published:** 2023-07-05

**Authors:** Jiaxin Liu, Taoyu Lin, Guanjun Liu, Xiaoxin Dong, Rui Min

**Affiliations:** ^1^School of Public Health, Tongji Medical College, Huazhong University of Science and Technology, Wuhan, China; ^2^The People’s Hospital of Suzhou New District, Suzhou, China; ^3^Institute of Health Services, Ningbo College of Health Sciences, Ningbo, China

**Keywords:** agitation, elderly adults, dementia, home-cared, Cohen-Mansfield agitation inventory

## Abstract

**Background:**

Agitation is common among older adults with dementia, negatively affecting their quality of life and their caregivers’. Since home care remains the dominant approach for older adults, this study investigates the risk factors for agitation in older adults with dementia in China.

**Methods:**

We perform a cross-sectional study of home-cared older adults with dementia in Ningbo, China, using 2020 data. We use a self-made questionnaire to investigate the risks of agitated behavior and its related factors. We perform descriptive, univariate, and regression analyses.

**Findings:**

We address 640 older Chinese adults; 42.8% of the sample exhibits one or more agitated behaviors. We find that basic health issues, such as activities of daily living (ADL), family support issues, such as Zarit Burden Interview (ZBI) scale and Family APGAR Questionnaire (APGAR), and behavioral awareness issues, such as fall and scald, significantly influence the occurrence of agitation behaviors (*p* < 0.05). Older adults with severe ADL disorder (*b* = 6.835, *β* = 0.196, *p* < 0.001), ZBI score of 67.00–88.0 (*b* = 10.212, *β* = 0.248, *p* = 0.005), severe APGAR disorder (*b* = 3.699, *β* = 0.100, *p* = 0.012) and a history of fall (*b* = 9.311, *β* = 0.199, *P* = <0.001) or scald (*b* = 9.288, *β* = 0.125, *p* = 0.002) are more likely to exhibit agitated behaviors.

**Interpretation:**

Agitated behavior in home-cared older adults with dementia are diverse and related to mental state, family support, and behavioral awareness issues. Caregivers, often family members, should be attentive to the needs of dementia patients and take active and effective measures to improve their quality of life. They should be aware of the causes and triggers of agitated behavior and take steps to reduce its occurrence.

## 1. Introduction

Dementia is a leading cause of disability in people older than 65 in China (Li et al., 2015; Charlson et al., 2016; Feigin et al., 2019). More than 55 million people live with dementia worldwide, and the number is expected to reach 78 million by 2030 (2022). In China, the incidence rate of dementia in older adults aged 60–69 years is 2.9%, and it reaches 31.9% in those over 90 years old (Jia et al., 2020). With the aging of the Chinese population, the number of elderly patients with dementia has increased (Wang et al., 2019). According to the latest statistics, in 2019, over 15.33 million Chinese people had dementia, and the number is expected to reach 45.33 million by 2050 (Nichols et al., 2022). Along with cognitive and functional decline, roughly five out of every six patients, including those who are home-cared, exhibit behavioral and psychological symptoms in dementia (BPSD) (Ismail et al., 2016). In addition, within 2 years of dementia diagnosis, 20% of those who were initially asymptomatic will develop symptoms, while 50–80% of those with significant symptoms will stay agitated for several months (Hendriks et al., 2015). Furthermore, at least 50% of older adults with dementia have severe BPSD on a monthly (Ryu et al., 2005). Agitated behavior is one of the most prevalent and difficult BPSD, causing feelings of helplessness and distress in families and formal caregivers (Givens et al., 2015), as well as being a strong predictor of poor quality of life (Wetzels et al., 2010).

Agitated behavior is defined as inappropriate verbal, vocal, or motor activity and encompasses physical and verbal aggression (Cohen-Mansfield and Billig, 1986). Among those with moderate or severe dementia, approximately 50% of patients experience behavioral symptoms every month, and 70–90% exhibit at least one or more behavioral symptoms during onset, the most frequent being physical non-agitated behavior (Seitz et al., 2010). A family survey has reported that the incidence of dementia agitation exceeds 50% (Hamel et al., 1990). The recurring hospitalization of the elderly with dementia is mostly due to the emergence of agitation behavior, which places a great load on both the elderly and their caregivers (Wolf et al., 2018; Yilmaz and Aşiret, 2021). When the agitated behavior of older adults with dementia is severe, it may lead to violent and aggressive behavior, threatening the safety of themselves and their caregivers. In addition, since almost all dementia patients rely on others for care during their illness, especially family caregivers (non-professional caregivers) (Liu et al., 2017), exploring factors related to agitated behavior in dementia patients is critical for the safety of older people with dementia and their caregivers.

Individual factors, human nature, and the physical environment have a cumulative influence on the onset and progression of agitation in the elderly with dementia. Understanding these influencing factors can assist caregivers in avoiding as much inducement and aggravation factors as feasible in order to effectively prevent the occurrence and development of agitation behavior. According to the existing research, the main six factors that cause agitation in older people with dementia are as follows: (1) Basic demographic factors: Cohen-Mansfield and Marx (1992) discovered a higher incidence of speech agitation in women compared to males, whereas Schreiner found no gender difference (Schreiner, 2001). For older adults with dementia receiving nursing home care, married older adults showed more aggressive behavior than unmarried e older adults (Cohen-Mansfield and Marx, 1992). In addition, other studies have shown a positive correlation between age and verbal arousal (Pelletier and Landreville, 2007). (2) Disease-related factors: dementia type and disease severity are also significant determinants of agitation. Most studies indicate that the severity of the disease and the degree to which cognitive function is impaired correlates with the severity of the agitated behavior of the older adults with dementia, particularly the physical aggressive, physical non-aggressive, and verbal aggressive behaviors (Robitaille et al., 2015). (3) Mental and psychological factors: BPSD include behavioral symptoms such as agitation, emotional symptoms such as depression and anxiety, and mental symptoms such as hallucinations and delusions, among which behavioral symptoms are often accompanied by emotional symptoms and mental symptoms (Bessey and Walaszek, 2019). Studies have shown that the severity of mental symptoms in older adults with dementia is positively correlated with the severity of aggressive behavior (Volicer et al., 2012). (4) Social and cultural environment factors: the social participation of older adults with dementia and the surrounding cultural environment are also important factors affecting agitation behavior. Lack of activity and communication disorder can lead to aggressive behavior (Talerico et al., 2002; Kong, 2005). (5) Physical environment factors: the physical environment around the older adults with dementia is another important dimension of the factors affecting agitation behavior (Kong, 2005). It is critical to maintain a stable, quiet, and pleasant atmosphere, as well as adequate stimuli, in order for older adults with dementia to feel at ease and familiar, which is critical for controlling agitation behavior. Understanding these influencing factors can assist caregivers in avoiding as much inducement and aggravation factors as feasible in order to effectively prevent the occurrence and development of agitation behavior. Furthermore, the majority of current studies focus on the agitation behavior of older adults with dementia in nursing homes, with few investigations on home-cared older adults with dementia. However, the statistics released by Alzheimer’s Disease International indicate that over 70% of older adults with dementia live at home (2022). Hence, the factors related to agitated behavior of dementia patients, especially those resorting to family support, should be explored. Reducing the occurrence of agitated behavior and improving the living conditions of patients with dementia are of utmost importance.

As the disease progresses, the majority of adults with dementia require care at home from family members (Van Den Wijngaart et al., 2007). Therefore, emotional support and life care from family members play a significant role in the occurrence of agitation in adults with dementia. This study investigated home-cared older adults with dementia and influencing factors of their aggressive behavior, which is crucial for reducing the incidence of dementia patients and improving the older adults’ healthy life quality.

## 2. Materials and methods

### 2.1. Study design

This study conducted a cross-sectional analysis of data collected from three communities in Ningbo City, Zhejiang Province, in 2020 to investigate the risk factors for agitation in home-cared older adults with dementia. All participants provided informed consent before the questionnaire. The local ethics committee approved this study. The study protocol and content were approved by the Research Ethics Committee of the Ningbo College of Health Sciences (NBWY-2019-012).

### 2.2. Participant

Inclusion criteria: a. being older than 60 as per the local population census; b. conforming to the Diagnostic and Statistical Manual of Mental Disorders, Fifth Edition (DSM-V) dementia diagnoses and being confirmed by clinicians; c. receiving home care; and d. willing to respond to the questionnaires.

Exclusion criteria: a. being under 60 years of age according to the local population census; b. Clinical Dementia Score (CDR) =0 or 0.5; c. receiving nursing home care or other non-home care.

### 2.3. Sampling

**Figure 1:**
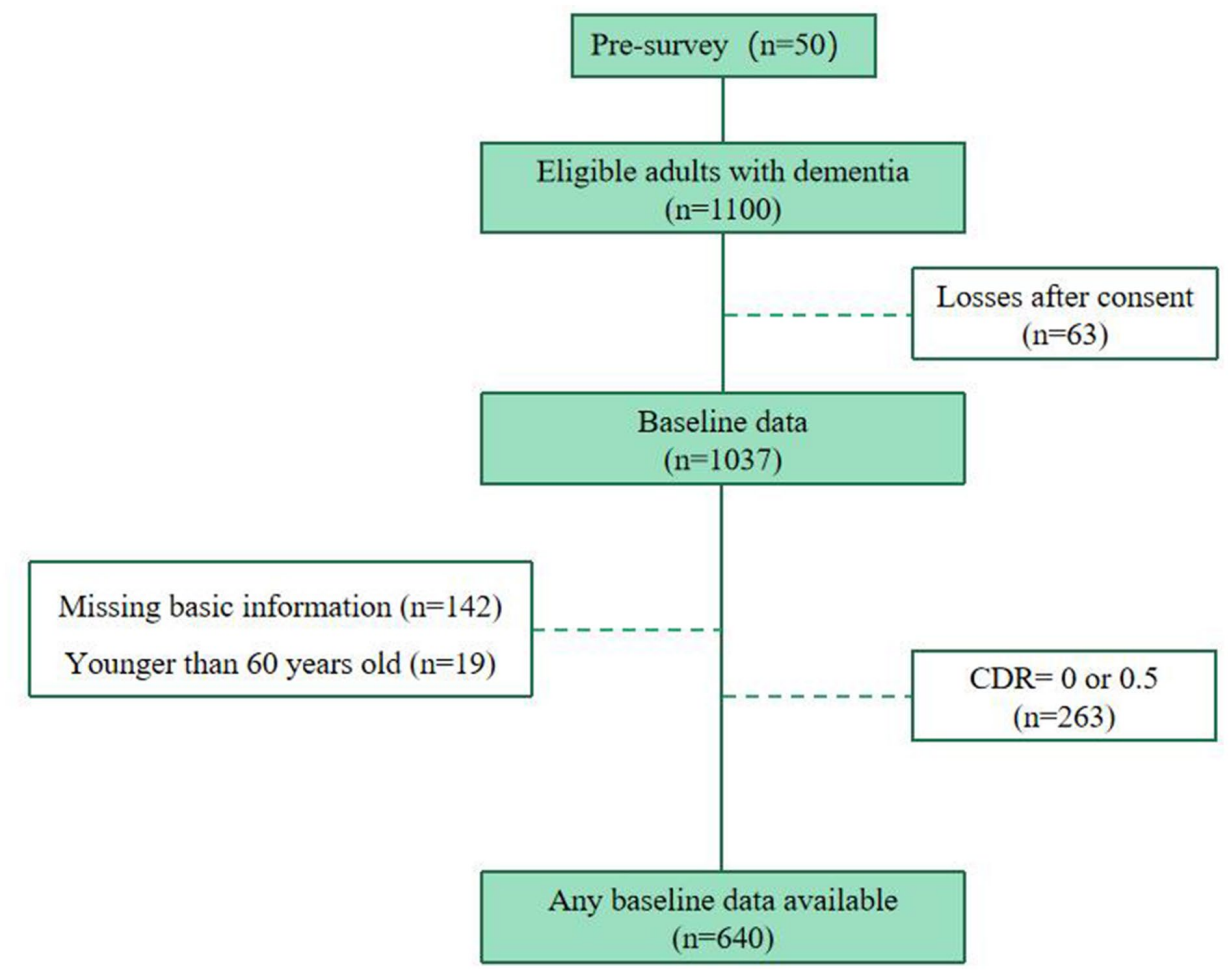
Baseline flow diagram of recruited older adults with dementia.

The sample size was calculated according to the survey sample size estimation formula for the current survey rate (Supplementary material). We removed questionnaire with missing age and sex, and missing CMAI score values of more than 10%. Finally, a total of 640 valid questionnaires were retained (Figure 1).

### 2.4. Study indicators

This study was conducted using a self-administered questionnaire based on a maturity scale. The questionnaire included three parts: the situation of older adults, assessment of agitation behavior, and potential risk factors (literature-learned). Specific questionnaire contents and indicators are in the (Supplementary material). The Cronbach’s α of the questionnaire is 0.888, whereas the Kaiser–Meyer–Olkin (KMO) was 0.911.

#### 2.4.1. Evaluation of agitation behavior

The Cohen Mansfield Agitation Inventory (CMAI) was used to evaluate agitation behavior of older adults with dementia. The CMAI has four dimensions and 29 items. Physical agitated behavior (12 items), physical non-agitated behavior (nine items), verbal agitated behavior (four items), and verbal non-agitated behavior (four items) (Cohen-Mansfield, 1986; Cohen-Mansfield et al., 1989; Lin et al., 2007). We rated the answers using a seven-point Likert Scale. The total score for agitated behavior ranged from 29 to 203 points; a total score ≥ 39 points indicated the existence of agitated behavior. The higher the score, the more severe the agitated behavior (Husebo et al., 2014). If at least two types of agitated behavior scored four points or above, or at least three scored three points or above, or at least four of scored two points or above, the behavior was considered agitated (Katz et al., 1963; Rabinowitz et al., 2005). The Cronbach’s α for the CMAI is 0.931, whereas the Kaiser–Meyer–Olkin (KMO) was 0.931.

#### 2.4.2. Basic health issues

We collected basic demographic information, including gender, age, marital status, education level of older adults, types of dementia and the number of chronic diseases.

Then, we used the activities of daily living (ADL) scale to evaluate the health status of participants. ADL has 10 items. Each item was rated as one to four, respectively. In line with the Barthel index score, more than 60 points represent good ability, 41–60 points denote moderate dysfunction and ≥ 40 points indicate severe dysfunction (Katz et al., 1963). The Cronbach’s α for the ADL is 0.925, whereas the Kaiser–Meyer–Olkin (KMO) was 0.865.

#### 2.4.3. Family support issues

Family support issues include number of children, education level of the caregiver, age of the caregiver and the caregiver’s knowledge of dementia.

We used the Zarit Burden Interview (ZBI) scale to assess caregivers’ feelings regarding the burden of caring for older adults with dementia at home. ZBI has four dimensions: caregiver health, mental state, and economic and social life. We considered 22 items, and each scored from zero to four points; a score of 21–40 and 41–60 points indicate no or mild burden and moderate or heavy burden, respectively (Zarit et al., 1980). The Cronbach’s α for the ZBI is 0.953, whereas the Kaiser–Meyer–Olkin (KMO) was 0.949.

Besides, we used the Family APGAR Questionnaire (APGAR) to measure the subjective satisfaction of older adults to family functions. The Family APGAR Questionnaire has five items and three possible responses (2, 1, 0) to each item. The total scores may range from 0 to 10 (low to high satisfaction with family function) (Smilkstein et al., 1982). The Cronbach’s α for the APGAR is 0.945, whereas the Kaiser–Meyer–Olkin (KMO) was 0.879.

#### 2.4.4. Behavioral awareness issues

We used the University of Washington Clinical Dementia Score (CDR) to assess the severity of Alzheimer’s disease (AD). The CDR is constructed from a semi-structured interview with the patient and a suitable informant and assesses impairment in each of six cognitive categories (Memory, Orientation, Judgment and Problem Solving, Community Affairs, Home and Hobbies, and Personal Care) using a five-point scale (Morris, 1993). From the six individual category ratings, clinical scoring rules determine the global CDR, with CDR 1, 2, or 3 indicating mild, moderate, or severe dementia (Morris, 1993). The Cronbach’s α for the CDR is 0.935, whereas the Kaiser–Meyer–Olkin (KMO) was 0.899.

In addition, we measured whether the elderly had fallen, aspirated, scalded and fallen out of bed in the last 3 months.

### 2.5. Statistical methods

We used SPSS 27.0 software for statistical description and data analysis. We employed the Mann–Whitney U tests and Kruskal-Wallis H tests to determine whether each variable affected the risk of agitation in home-cared older adults with dementia (*p* < 0.05). After identifying the relevant factors as independent variables, we employed the CMAI score as the dependent variable. We used a multiple linear model to evaluate correlations with agitation (*p* < 0.05). We set the test’s significance level at *α* = 0.05 (Miller and Ulrich, 2019).

## 3. Results

### 3.1. Occurrence of four types of agitated behavior among home-cared older adults with dementia

**Figure 2:**
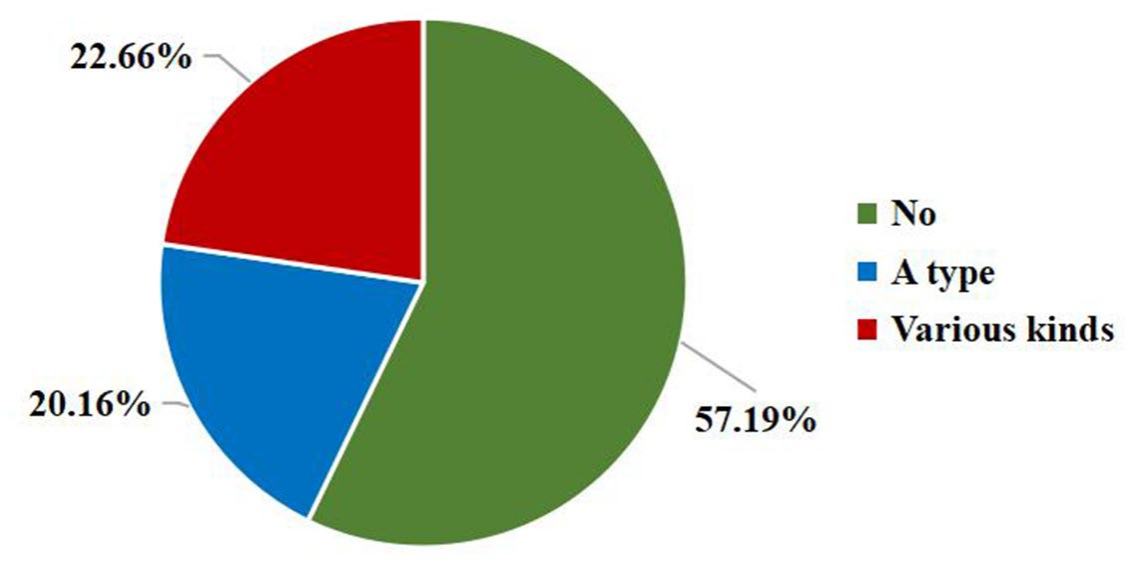
The occurrence of four types of agitated behavior in the older adults with dementia.

In this study, 33.3% home-cared older adults had mild dementia, 29.7% had moderate dementia, and 37% had severe dementia (Table 1). We measure the risk of four types of agitated behavior in older adults with dementia using CMAI. The incidence of physical non-agitated behavior is the highest (37.0%), followed by physical agitated behavior (20.9%), verbal non-agitated behavior (13.0%), and verbal agitated behavior (10.5%). Moreover, as shown in Figure 2, approximately one-fourth of older adults with dementia exhibited two or more types of agitation simultaneously.

**Table 1:** One-factor analysis of risk factors associated with agitated behavior.

Variable		N (%)	Median	Test statistic	*p*-value
Basic health issues					
Gender	Males	297 (46.4%)	36.0 (29.0 ~ 50.0)	51497.500	0.808*
	Females	343 (53.6%)	35.0 (30.0 ~ 49.0)		
Age (in years)	60–74	204 (31.9%)	38.0 (31.0 ~ 53.0)	16.613	**<0.001** ^**✢**^
	75–89	361 (56.4%)	35.0 (29.0 ~ 48.0)		
	Above 90	75 (11.7%)	33.0 (29.0 ~ 40.0)		
Marital status	Married	308 (48.4%)	35.0 (29.0 ~ 48.5)	0.945	0.331*
	Single	328 (51.6%)	36.0 (30.0 ~ 50.0)		
Educational level of the elderly	Illiteracy	174 (27.2%)	39.0 (31.0 ~ 55.0)	15.180	**0.002** ^**✢**^
	Primary	247 (38.6%)	36.0 (29.0 ~ 51.0)		
	Middle	197 (30.8%)	33.0 (29.0 ~ 42.0)		
	University and above	22 (3.4%)	35.5 (29.0 ~ 51.0)		
Types of dementia	Alzheimer’s disease	391 (71.2%)	35.0 (29.0 ~ 51.0)	6.245	0.100^✢^
	Vascular dementia	33 (6.0%)	38.0 (30.0 ~ 57.0)		
	Mixed dementia	68 (12.4%)	39.5 (31.5 ~ 51.5)		
	Others	57 (10.4%)	33.0 (29.0 ~ 43.0)		
Number of chronic diseases	No	119 (18.7%)	33.0 (29.0 ~ 44.0)	7.341	0.025^✢^
	A type	173 (27.1%)	36.0 (30.0 ~ 52.0)		
	Various kinds	346 (54.2%)	36.0 (30.0 ~ 50.0)		
Activities of daily living	Very mild	103 (16.1%)	29.0 (29.0 ~ 32.0)	93.348	**<0.001** ^**✢**^
	Mild	189 (29.6%)	37.0 (32.0 ~ 50.0)		
	Moderate	97 (15.2%)	43.0 (34.0 ~ 57.0)		
	Severe	250 (39.1%)	37.0 (29.0 ~ 53.0)		
Family support issues					
Number of children	0	60 (9.6%)	37.5 (32.0 ~ 51.0)	8.656	0.034^✢^
	1	145 (23.2%)	33.0 (29.0 ~ 46.0)		
	2	207 (33.1%)	35.0 (29.0 ~ 49.0)		
	Above3	214 (34.2%)	37.0 (30.0 ~ 53.0)		
Zarit burden interview	≤22.00	19 (3.0%)	29.0 (29.0 ~ 35.0)	65.740	**<0.001** ^**✢**^
	23.00–44.00	176 (27.5)	32.0 (29.0 ~ 39.0)		
	45.00–66.00	283 (44.3%)	36.0 (30.0 ~ 46.0)		
	67.00–88.00	145 (22.7%)	49.0 (31.0 ~ 61.0)		
	Above89.00	16 (2.5%)	48.0 (32.0 ~ 72.5)		
Educational level of the caregivers	Illiteracy	39 (6.3%)	41.0 (30.0 ~ 60.0)	13.695	**0.003** ^**✢**^
	Primary	220 (35.4%)	35.0 (29.0 ~ 46.5)		
	Middle	285 (45.8%)	37.0 (29.0 ~ 43.0)		
	University and above	78 (12.5%)	33.0 (29.0 ~ 43.0)		
Age of the caregivers	Under 44	115 (18.1%)	35.0 (29.0 ~ 45.0)	21.792	**<0.001** ^**✢**^
	45–59	274 (43.2%)	37.0 (31.0 ~ 53.0)		
	60–74	159 (25.1%)	36.0 (30.0 ~ 53.0)		
	Above 75	86 (13.6%)	31.0 (29.0 ~ 43.0)		
Caregiver knowledge	Not understood	166 (27.0%)	33.5 (30.0 ~ 51.0)	1.721	0.423^✢^
	General	248 (38.8%)	35.0 (29.0 ~ 49.0)		
	See	201 (31.4%)	39.0 (30.0 ~ 50.0)		
APGAR	Mild	317 (50.2%)	32.0 (29.0 ~ 41.0)	66.282	**<0.001** ^**✢**^
	Moderate	119 (18.8%)	38.0 (31.0 ~ 48.0)		
	Severe	196 (31.0%)	43.0 (34.0 ~ 56.0)		
Behavioral awareness issues					
Clinical dementia rating	Mild	213 (33.3%)	35.0 (29.0 ~ 47.0)	3.658	0.161^✢^
	Moderate	190 (29.7%)	34.0 (29.0 ~ 50.0)		
	Severe	237 (37.0%)	37.0 (31.0 ~ 50.0)		
Fall	No	534 (83.7%)	34.0 (29.0 ~ 45.0)	42501.500	<0.001*
	Yes	104 (16.3%)	53.0 (41.0 ~ 65.0)		
Aspiration	No	549 (88.7%)	34.0 (29.0 ~ 46.00)	27261.500	<0.001*
	Yes	70 (11.3%)	52.5 (38.0 ~ 61.0)		
Scald	No	575 (94.3%)	35.0 (29.0 ~ 47.0)	15848.000	<0.001*
	Yes	35 (5.7%)	58.0 (46.0 ~ 73.0)		
Falling out of the bed	No	569 (93.3)	35.0 (29.0 ~ 47.0)	18446.000	<0.001*
	Yes	41 (6.7%)	58.0 (44.0 ~ 72.0)		

Data are expressed as median (interquartile range). *P*-value < 0.05 indicates stat. *Mann-Whitney *U*-tests; ^✢^Kruskal-Wallis H tests. Bold values are statistically significant.

### 3.2. Associated factors of agitated behavior risk among home-cared older adults with dementia

The results of the one-factor analysis (Table 1) show that, in terms of the basic health issues, age, level of education, ADL of home-cared older adults with dementia were associated to the occurrence of agitation behavior (*p* < 0.05) (Table 1). In terms of the family support issues, ZBI, the education level of caregivers, the age of caregivers and APGAR were associated with agitated behavior (*p* < 0.05) (Table 1). Furthermore, in terms of the behavioral awareness issues, home-cared older adults with dementia who had fallen, aspirated, scalded, and fallen out of bed in the last 3 months were likely to have agitated behavior (*p* < 0.05) (Table 1).

### 3.3. Regression analysis of factors related to agitated behavior risk among home-cared older adults with dementia

We employed the basic health issues, family support issues and behavioral awareness issues as independent variable, and the CMAI score as dependent variable. We used a multiple linear model to evaluate correlations.

The regression analysis results (Table 2) show that, in terms of the basic health issues, the home-cared older adults with mild ADL disorder (*b* = 5.197, *β* = 0.140, *p* = 0.007), moderate ADL disorder (*b* = 11.087, *β* = 0.228, *p* < 0.001), and severe ADL disorder (*b* = 6.835, *β* = 0.196, *p* < 0.001) were more likely to exhibit agitated behavior than the older adults with dementia who received better home care with ADL. Moreover, older adults with a high school education (*b* = −4.719, *β* = −0.129, *p* = 0.004) were less likely to exhibit agitated behavior than the illiterate.

**Table 2:** Multiple disorderly regression of the risk factors associated with agitated behavior.

Variable	Unstandardized coefficients	Standardized coefficients	*P*-value
Basic health issues			
75–89 years old of the elderly	−4.127 ± 1.360	−0.120	**0.003**
Above 90 years old of the elderly	−9.272 ± 2.169	−0.175	**<0.001**
Activities of daily living = 2.00	5.197 ± 1.912	0.140	**0.007**
Activities of daily living = 3.00	11.087 ± 2.258	0.228	**<0.001**
Activities of daily living = 4.00	6.835 ± 1.897	0.196	**<0.001**
Primary school education level of the elderly	−3.019 ± 1.545	−0.087	0.051
Middle school education level of the elderly	−4.719 ± 1.647	−0.129	**0.004**
University and above education level of the elderly	−0.597 ± 3.407	−0.006	0.861
Family support issues			
Zarit burden interview = 23.00–44.00	−0.409 ± 3.402	−0.011	0.905
Zarit burden interview = 45.00–66.00	2.666 ± 3.402	0.078	0.434
Zarit burden interview = 67.00–88.00	10.212 ± 3.591	0.248	**0.005**
Zarit burden interview = Above89.00	8.577 ± 5.089	0.077	0.092
Primary school education level of the caregiver	−1.306 ± 2.394	−0.037	0.586
Middle school education level of the caregiver	1.909 ± 2.390	−0.056	0.425
University and above education level of the caregiver	−3.071 ± 2.885	0.060	0.288
45–59 years old of the caregiver	−0.0.071 ± 1.662	−0.002	0.966
60–74 years old of the caregiver	0.942 ± 1.786	0.025	0.598
Above 75 years old of the caregiver	−2.867 ± 2.541	−0.045	0.260
Moderate family dysfunction	3.827 ± 1.617	0.088	**0.018**
Severe family dysfunction	3.699 ± 1.476	0.100	**0.012**
Behavioral awareness issues			
Fall	9.311 ± 1.960	0.199	**<0.001**
Aspiration	−1.095 ± 2.208	−0.496	0.620
Scald	9.288 ± 3.042	0.125	**0.002**
Falling out of the bed	4.266 ± 3.092	0.061	0.168

Bold values are statistically significant.

Then, in terms of the family support issues, home-cared older adults with ZBI score of 67.00–88.0 (*b* = 10.212, *β* = 0.248, *p* = 0.005) were more likely to exhibit agitated behavior than those with a ZBI score of 22.0 or lower. Additionally, older adults with moderate (*b* = 3.827, *β* = 0.088, *p* = 0.018) or severe family dysfunction (*b* = 3.699, *β* = 0.100, *p* = 0.012) were more likely to exhibit agitated behavior than those with healthy family function.

Furthermore, in terms of the behavioral awareness issues, older adults with dementia who had fallen (*b* = 9.311, *β* = 0.199, *P* = <0.001) or scalded (*b* = 9.288, *β* = 0.125, *p* = 0.002) in the past 3 months were more likely to have agitated behavior.

## 4. Discussion

After a multi analysis of home-cared older adults with dementia in Ningbo, China, we found that among the 640 elders, 37% have severe dementia, 37% show physical non-agitated behavior, and almost 1/4 exhibit two or more types of agitation simultaneously. The results of this study indicates that the basic health issues, family support issues and behavioral awareness issues of home-cared older adults with dementia have an impact on the occurrence of their agitated behavior. Further analysis of these influencing factors is of great significance for the study of home care for the older adults with dementia.

### 4.1. Analysis of the current situation in home-cared older adults with dementia

This study indicated that home-cared older adults with dementia were slightly more likely to exhibit two or more agitated behaviors (22.7%) than to exhibit only one agitated behavior (20.2%). In addition, among the four agitated behavior, the incidence of physical non-agitated behavior was the highest (37.0%), followed by physical agitated behavior (20.9%), which may be owing to the fact that family caregivers are more likely to observe volatile, transient, and minor behaviors, such as physical non-agitated behaviors such as repetitive movements and verbal non-agitated behaviors such as repetitive sentences, than professional nursing staff, such as nurses, who contribute attention to behaviors that affect nursing work or daily management, such as physical agitated behaviors (Dukas, 2020).

Physical non-agitated behavior is associated with the older adults’ incapacity to express their needs or lead a monotonous lifestyle, reflecting the older adults’ need for social opportunities or physical exercise (Altunöz et al., 2015). Verbal non-agitated behavior is linked with the caregiver providing care without communicating with the patient, and repeated sentences or inquiries had been most prevalent, consistent with memory loss in patients with cognitive dysfunction (Isaac et al., 2021). Therefore, family caregivers of home-cared older adults with dementia should pay more attention to the social requirements and mutual communication of older adults.

### 4.2. The impact of basic health issues on agitated behavior in home-cared older adults with dementia

Due to memory, learning, thinking, spirit, and other aspects of the disorder, older adults with dementia frequently lose the ability to care for themselves. Previous studies have shown that the occurrence and persistence of agitation behavior in dementia patients is significantly related to the decline of daily living ability (Beck et al., 1998). In this study, we used the ADL scale to assess the daily living ability of older adults, and the results of multiple regression also showed that the lower the ADL of home-cared dementia patients, the higher the agitation score. The possible reason is that home-cared older adults with dementia who are better at daily living have more opportunities to interact with others and engage in physical activity, but the loss of daily living abilities makes it difficult for older adults to comprehend environmental stimulation, causing agitation (Lin et al., 2009). This finding reveals that as the disease progresses, home-cared older adults with dementia will require increasing assistance with activities of daily living, caregivers should communicate with the older adults with more patience and actively engage them in necessary physical activity and socialization.

### 4.3. The effects of ZBI and APGAR on agitated behavior in home-cared older adults with dementia

In China, due to the inequalities of the current medical security system and the impact of traditional family culture, home-based care has always played an essential role. In this study, about 50.1% of home-care providers for older adults with dementia are their spouses and children. When older adults with dementia reside with their families, family interventions (particularly family caregivers) can influence the disease’s progression or improvement to some extent. As the primary healthcare provider for older adults, family caregivers are responsible for providing a variety of nursing and support services. Long-term and intensive care will bring great burden to caregivers (Xiao et al., 2014). Previous research has demonstrated that the living conditions, care-giving abilities, and physical and mental health of caregivers have a direct impact on the quality of life and prognosis of older adults with dementia (Norton et al., 2013; Park et al., 2015). In this study, we use ZBI to assess the burden of care-giving across four dimensions: health, mental state, economic difficulties, and social life. The results indicated that dementia patients with a ZBI greater than 67 are more likely to exhibit agitated behavior than those with a ZBI of less than 23. Family can provide practical support for the elderly, but an increase in family burden will have a negative effect on the progression of dementia in the elderly. Continuous support and assistance for family caregivers can reduce care-giving stress and improve their quality of life, as well as improve the health of older adults.

Additionally, for home-cares, daily life support is essential, but emotional support from family members should also be considered. In the context of the Chinese family pension, family is the primary or even sole focus of the social activity of older adults in their homes. The reactions of family caregivers to the emotions of the elderly, as well as how they interact with one another, have a significant impact on the emotional fluctuations of the elderly. In this research, we find that compared to older adults with well APGAR, those with moderate or severe APGAR were more likely to exhibit agitated behavior. Previous studies have also found that almost all variables of family dynamics (family satisfaction) are significantly associated with all variables of mental health for caregivers, for example, caregivers’ mental health is stronger when their family functions well (Sutter et al., 2014). As a result, family caretakers should pay more attention to methods and encouragement when interacting with the elderly. For example, when the elderly desire to participate in new activities or develop, family members should give their full approval and support.

### 4.4. Fall and scald are more likely to trigger agitated behavior in home-cared older adults with dementia

This study showed that older adults with dementia who had fallen or scalded in the last 3 months are more likely to have agitated behavior, which is probably because the patient’s physical injury will affect the patient’s mental state, and then cause the occurrence of provocative behavior. For example, approximately 5–15% of the elderly will sustain brain injuries, soft tissue contusions, dislocations, and other similar injuries as a result of falls (Katz et al., 2004). Furthermore, older adults with a high risk of falls and scalds have sequelae such as mobility disorders induced by some cerebrovascular diseases, which will also contribute to the occurrence of agitated behavior. Previous studies have also shown that there is a correlation between agitated behavior and the occurrence of falls in dementia patients (Katz et al., 2004; Dong et al., 2022). As a result, during the care process, we must also attach importance to avoid the occurrence of high-risk behavior that will harm the older adults’ physical health. For instance, the caregivers should inspect the indoor furniture and lighting every week, increase the bar and lighting in the indoor area where the older adults frequently engage in activities to reduce the risk of falls, adjust the height and placement of the kettle to reduce the risk of scalds, and more. Additionally, the older adults with dementia should avoid going to crowded locations and being outside when it is raining or snowing.

### 4.5. Limitations and prospects

Some limitations can be pointed from this study. Firstly, our study is a cross-sectional study and only surveyed in one city, which makes the representativeness of the sample may be insufficient. Secondly, we have only explored basic health issues, family support issues, behavioral awareness issues, and there is insufficient research on the living environment of dementia patients.

As we know, agitated behaviors are diverse and closely related to mental state, family support and caregivers’ burden. The results of this study, demonstrating that the occurrence of agitated behavior is related to the ADL, ZBI, APGAR. For the home-cared older adults with dementia, their caregivers, such as relatives and adult children, should pay attention to the company of older adults with dementia, the causes and rules of dementia older adults’ anxiety behaviors, and take active and effective coping ways to improve the quality of life of older adults with dementia.

## Data availability statement

The raw data supporting the conclusions of this article will be made available by the authors, without undue reservation.

## Author contributions

RM planned the study and the overall analysis method. JL and TL substantially contributed to acquisition and interpretation of data and writing of the manuscript. GL and XD contributed to data analysis and data collation. All authors contributed to the article and approved the submitted version.

## Funding

This study was supported by the Fundamental Research funds for the Central Universities (HUST:2020kfyXJJS057) and the Natural Science Foundation of Zhejiang Province (LQ20H260006).

## Conflict of interest

The authors declare that the research was conducted in the absence of any commercial or financial relationships that could be construed as a potential conflict of interest.

## Publisher’s note

All claims expressed in this article are solely those of the authors and do not necessarily represent those of their affiliated organizations, or those of the publisher, the editors and the reviewers. Any product that may be evaluated in this article, or claim that may be made by its manufacturer, is not guaranteed or endorsed by the publisher.
