# Supplementary material for: Risk factors for agitation in home-cared older adults with dementia: evidence from 640 elders in East China
Source: Front Neurosci. 2023 Jul 5;17:1189590. doi: 10.3389/fnins.2023.1189590 (PMC10354277; doi:10.3389/fnins.2023.1189590)
Supplement: Supplementary file 1 [file Data_Sheet_1.docx]

Supplementary Material

**Risk factors for agitation in home-cared older adults with dementia: Evidence from 640 elders in East China**

**Jiaxin Liu, Rui Min***

*** Correspondence:** Corresponding Author: Rui Min email:[ruimin0801@163.com](mailto:ruimin0801@163.com)

1. **Sampling**

We surveyed the elderly in Ningbo, screening individuals with a definite diagnosis or self-complaint of dementia. The sample size was calculated according to the survey sample size estimation formula for the current survey rate, as follows:

$$n=\frac{Z_{a/2}^{2}\cdot P\left( 1-P \right)}{\delta^{2}}$$

where *P* is the sampling rate, and Z is the z-boundary value of a normal variable corresponding to the bilateral tail area under the standard normal distribution. The significance level is set to 0.05; hence, $Z_{a/2}$=1.96. The allowable error was set to 0.15 P.

Previous studies showed that the incidence of agitation in home-cared older adults with dementia in China in recent years has ranged between 50% and ­90%. We calculated the sample size based on the 50% (*P*) risk incidence rate to ensure representativeness. The dementia older adults and caregivers were 171. Considering a 20% loss to follow-up rate, the total sample size was at least 214. Finally, 1037 questionnaires were collected, excluding those with more than 10% missing information (142) , those from individuals younger than 60 years old (19) and those with Clinical Dementia Score (CDR)= 0 OR 0.5. A total of 640 valid questionnaires were retained.

1. **Quality control**

Relevant training was provided before and during the project, with a cumulative duration of three hours. We recruited medical professionals as surveyors. These professionals were invited to explain the questionnaire items, its contents, precautions of the scale, and investigation skills, providing demonstrations. A face-to-face approach was employed to describe the survey's purpose and precautions to older adults and their families. The survey emphasized the principle of confidentiality and was conducted orally. The investigators filled out the questionnaires, and two people in each group conducted the survey. Before the statistical analysis, we organized the data and eliminated invalid questionnaires; after the data entry phase, we double-checked the data to ensure the data entry's accuracy.

1. **Study indicators**

| Indicators | type |
| --- | --- |
| Basic health issues | |
| Gender | [categorical variable]  1=male, 2=female |
| Age (in years) | [categorical variable]  1=60-70, 2=70-79, 3=80-89, 4=90-99 |
| Marital status | [categorical variable]  1=spouse present, 2=divorced, 3=widowed, 4=Never married |
| Educational level of the elderly | [categorical variable]  1=illiteracy, 2=primary school, 3=middle school, 4=University and above |
| Types of dementia | [categorical variable]  1=Alzheimer's disease, 2=Vascular dementia, 3=Mixed dementia, 4=others |
| Number of chronic diseases | [categorical variable]  1=No, 2=A type, 3=Various kinds |
| Activities of daily living | [categorical variable]  1=life self-care, 2=Mild dysfunction, 3=Moderate dysfunction, 4=Severe dysfunction |
| Family support issues | |
| Number of children | [categorical variable]  1=0, 2=1, 3=2, 4=Above 3 |
| Zarit Burden Interview | [categorical variable]  1=<= 22.00, 2=23.00 - 44.00, 3=45.00 - 66.00, 4=67.00 - 88.00, 5=Above 89.00 |
| Educational level of the caregivers | [categorical variable]  1=illiteracy, 2=primary school, 3=middle school, 4=University and above |
| Age of the caregivers | [categorical variable]  1=under 44, 2=45-59, 3=60-74, 4=above 75 |
| Caregiver knowledge | [categorical variable]  1=not understood, 2=General, 3=See |
| The Family APGAR Questionnaire | [categorical variable]  1=Mild, 2=Moderate, 3=Severe |
| Behavioural awareness issues | |
| Clinical dementia rating | [categorical variable]  1=Mild, 2=Moderate, 3=Severe |
| Fall | [categorical variable]  1=No, 2=Yes |
| Aspiration | [categorical variable]  1=No, 2=Yes |
| Scald | [categorical variable]  1=No, 2=Yes |
| Falling out of the bed | [categorical variable]  1=No, 2=Yes |

1. **Questionnaires**

**Safety Risk Questionnaire of Home Care for Mentally Disabled Elderly**

**Dear old people and caregivers:**

**Hello!**

**This survey is mainly to comprehensively understand the possible security risks of home care for the elderly with dementia, as well as the burden of caregivers, and provide the basis for the government to formulate pension service policies, in order to improve the care service level of the elderly with dementia, and ultimately improve the life quality and happiness of the elderly. Hope you can truthfully fill in the relevant content, the personal information involved, we will strictly confidential! Thank you for your participation! Have a happy life!**

**Project group**

**A Basic situation** **of the elderly**

**A1 Sex of the elderly [Investigator Observation]:**

1. Male 2. Female

**A2 Age of the elderly: years (based on the past birthday)**

**A3 The educational level of the elderly:**

1. No schooling 2. Primary school 3. Junior high school

4. High school/technical secondary school/vocational school

5. College degree 6. Bachelor degree and above

**A4 Current marital status of the elderly:**

1. Spouse, age 2. Divorce 3. Widows 4. Never married

**A5 Current situation of children of the elderly:**

1. Children 2. No children

**A6 Who does the old man live with now? (Multiple-choice questions)**

1. Living alone 2. Spouses 3. Children 4. Other relatives 5. Nurse 6. Other

**A7 What is the average monthly income of the elderly?**

1. ≤¥1000 2. ¥1001~ ¥3000 3. ¥3001~ ¥5000 4. ¥5001 ~ ¥7000 5. ≥ ¥7001

**A8 What medical insurance does the elderly in enjoy?**

1. No 2. Basic Medical Insurance for Urban Employees

3. Basic Medical Insurance for Urban and Rural Residents (Integration of Basic Medical Insurance for Urban Residents and New Rural Cooperative Medical Scheme)

4. Public Health (Cadre Health, etc.) 5. Commercial Medical Insurance 6. Other

**A9. Is the diagnosis of dementia in elderly clear?**

1.No 2.Yes

**A9.1 Which of the following types is clearly diagnosed?**

1. Alzheimer’ s disease 2. Vascular dementia 3. Mixed dementia

4. Ravel’ s dementia 5. Other types

**A10. Does elderly adults take anti - dementia drugs?**

1. not taking 2. one kind a day 3. many kinds a day 4. one kind a week

5. many kinds a week 6. unclear.

**A11. What chronic diseases do the A11 elderly suffer from? (Multiple-choice questions)**

1. No 2.other mental illnesses (depression, schizophrenia, etc.) 3. Hypertension 4. Diabetes 5. Cardiovascular and cerebrovascular diseases 6. Cataract / glaucoma

7. Gastric diseases 8. Osteoarthritis 9. Chronic pulmonary diseases 10. Asthma 11. Malignant tumors 12. Reproductive system diseases 13. Other chronic diseases

**A12 What are the following daily activities of the elderly? [Investigator: Select conformity option in each item]**

| **item** | **1 complete independence** | **2 Partial help** | **3 Great help** | **4 Complete dependences** |
| --- | --- | --- | --- | --- |
| 1. Food intake | 10 | 5 | 0 | ---- |
| 2. Bathing | 5 | 0 | ---- | ---- |
| 3. Modification (washing, brushing, shaving, combing) | 5 | 0 | ---- | ---- |
| 4 Wearing (shoelaces, buttons, etc.) | 10 | 5 | 0 | ---- |
| 5. Control of defecation | 10 | 5 (out of control < 1 time per week) | 0 (out of control) | ---- |
| 6. Control urine | 10 | 5 (out of control < 1 time per week) | 0 (out of control) | ---- |
| 7. Toilet (clean, tidy, flush) | 10 | 5 | 0 | ---- |
| 8. Transfer of beds and chairs | 15 | 10 | 5 | 0 |
| 9. Walking 45 miters flat | 15 | 10 | 5 | 0 |
| 10. Up and down stairs | 10 | 5 | 0 | ---- |

**A13 What is the intelligence status of the elderly? [ Investigator: Select conformity option in each item]**

| item | Dementia-free  CDR0 | Suspicious dementia  CDR0.5 | Mild dementia  CDR1.0 | Moderate dementia  CDR2.0 | Severe dementia  CDR3.0 |
| --- | --- | --- | --- | --- | --- |
| Memory | 1. Forgetfulness without memory impairment or only mildly unstable | 2. Mild and sustained amnesia; partly recalling things | 3. Moderate memory impairment; forgetting about recent events is prominent and impairs the memory of daily activities | 4. Severe memory impairment; remember the familiar things of the past, and the new things quickly forget | 5. Severe memory loss; memory with only fragments |
| Directional force | 1. Completely correct orientation | 2. Completely correct orientation except for minor difficulties in time orientation | 3. Moderate difficulty in timing; directional to inspection sites; it may lose orientation in other locations | 4.Time orientation has serious difficulties; frequent location deorientation | 5. Person orientation only |
| Judgment + problem-solving ability | 1.It can well solve daily problems, handle affairs and finance; good judgment | 2.Slight defect | 3. Moderate difficulties; social judgment is usually preserved | 4.Serious damage; social judgment is usually impaired | 5. Cannot judge or solve problems |
| Social affairs | 1. The same level of independence in work, shopping and community activities as in the past | 2. Minor damage to these activities | 3. Although they also participate, they cannot carry out these activities independently; occasionally normal | 4. Outdoor activities cannot be conducted independently; but it can be taken to outdoor activities | 5. Outdoor activities cannot be conducted independently; the illness cannot be brought to outdoor activities |
| Family + hobbies | 1. Interest in family life, hobbies and intellectual needs is well maintained | 2. Minor damage | 3. Mild obstacles to family activities, abandonment of difficult household tasks, abandonment of complex hobbies and interests | 4. Be able to do simple housework only and maintain very limited scope and level of interest | 5. Loss of meaningful family activities |
| Personal | 1. Fully capable of self-care |  | 3. Need for oversight | 4. Need for assistance in dress, hygiene, personal financial custody | 5.Personal care needs a lot of help; frequent incontinence |

**B. Risk factor assessment**

**[Investigator: Select conformity option in each item]**

| **B1 fall assessment** | **1** | **2** | **3** | **4** | **5** |
| --- | --- | --- | --- | --- | --- |
| **1. Have you fallen in the last three months?** | No | 1–2 times | 3–4 times | 5–6 times | more than 6 times |
| 2. Are there more than one different system medical diagnosis? | No | 2 | 3 | 4 | 4 or more |
| 3. Do you use walking aids? | Not used | Activities supported by people | Using crutches | Using walk aid | Walk by furniture |
| 4. Whether intravenous infusion or heparin lock? | None | Very few | Sometimes | Frequently | Always |
| 5. Is gait normal? | Normal | Bed inactivity | little weak lower limb | Very weak legs | Disability or dysfunction |
| 6. Will you overestimate your ability? | None | Very few | Sometimes | Frequently | Always |

**B3 Has any aspiration occurred in the past three months?**

1. None 2. 1-2 times 3. 3-4 times 4. 5-6 times 5. More than 6 times

**B4 Has the elderly been scalded in recent three months?**

1. None 2. 1-2 times 3. 3-4 times 4. 5-6 times 5. More than 6 times

**B5 Has the elderly fallen into bed in the past three months?**

1. None 2. 1-2 times 3. 3-4 times 4. 5-6 times 5. More than 6 times

**B6 Passion Behavior Assessment (CMAI)** [Note: A review of the past two weeks of observed acts of agitation, according to the frequency of occurrence ' never appeared a specific act of agitation ' to ' several times per hour the act of agitation ' in turn 1 to 7 points.]

| **item** | None | 1 in nearly 2 weeks | 1 per week | Multiple weekly | Almost every day | Multiple times a day | Multiple per hour |
| --- | --- | --- | --- | --- | --- | --- | --- |
| 1.Walking or wandering purposeless | 1 | 2 | 3 | 4 | 5 | 6 | 7 |
| 2.Inappropriate dress or coat | 1 | 2 | 3 | 4 | 5 | 6 | 7 |
| 3.Spit at will (including meals) | 1 | 2 | 3 | 4 | 5 | 6 | 7 |
| 4.curse others or intimidate or insult others in words | 1 | 2 | 3 | 4 | 5 | 6 | 7 |
| 5.Irrational requests for attention or assistance | 1 | 2 | 3 | 4 | 5 | 6 | 7 |
| 6.Repeated speaking or questioning | 1 | 2 | 3 | 4 | 5 | 6 | 7 |
| 7.Strike or self | 1 | 2 | 3 | 4 | 5 | 6 | 7 |
| 8.Kicking or objects | 1 | 2 | 3 | 4 | 5 | 6 | 7 |
| 9.Catch others or themselves or objects. | 1 | 2 | 3 | 4 | 5 | 6 | 7 |
| 10.Push others away | 1 | 2 | 3 | 4 | 5 | 6 | 7 |
| 11.Tossing items (including food) or sweeping them off the table | 1 | 2 | 3 | 4 | 5 | 6 | 7 |
| 12.An unusual sound (strange laughter or crying) | 1 | 2 | 3 | 4 | 5 | 6 | 7 |
| 13.Scream, shout or howl | 1 | 2 | 3 | 4 | 5 | 6 | 7 |
| 14.Bite or object | 1 | 2 | 3 | 4 | 5 | 6 | 7 |
| 15.To be close to or hold on to others | 1 | 2 | 3 | 4 | 5 | 6 | 7 |
| 16.Unprovoked departure or unauthorized access to other places | 1 | 2 | 3 | 4 | 5 | 6 | 7 |
| 17.deliberate fall | 1 | 2 | 3 | 4 | 5 | 6 | 7 |
| 18.Complaints or complaints | 1 | 2 | 3 | 4 | 5 | 6 | 7 |
| 19.Negative | 1 | 2 | 3 | 4 | 5 | 6 | 7 |
| 20.Eating and drinking non-food items | 1 | 2 | 3 | 4 | 5 | 6 | 7 |
| 21.Injuring oneself or others | 1 | 2 | 3 | 4 | 5 | 6 | 7 |
| 22.Unproper handling of things (random search of drawers, unauthorized access to other people 's objects or untouchables) | 1 | 2 | 3 | 4 | 5 | 6 | 7 |
| 23.Hidden objects | 1 | 2 | 3 | 4 | 5 | 6 | 7 |
| 24.Storage or collection of excessive or improper items | 1 | 2 | 3 | 4 | 5 | 6 | 7 |
| 25.Torn or destroyed objects / property | 1 | 2 | 3 | 4 | 5 | 6 | 7 |
| 26.Repeated actions (shaking body, rubbing body or object, tapping objects, and ripping skin) | 1 | 2 | 3 | 4 | 5 | 6 | 7 |
| 27.Oral requests | 1 | 2 | 3 | 4 | 5 | 6 | 7 |
| 28.Sexual behaviors | 1 | 2 | 3 | 4 | 5 | 6 | 7 |
| 29.Restless or restless | 1 | 2 | 3 | 4 | 5 | 6 | 7 |

**C Assessment of the surrounding living environment**

|  |  |  |  |  |  |
| --- | --- | --- | --- | --- | --- |
| 1.Can the home environment reduce the security risks of the elderly and improve their sense of security? | Not at all | Not very competent | General | Almost | Completely able |
| 2.Can the home environment strengthen the elderly 's clear identification of living space, time and sociality? | Not at all | Not very competent | General | Almost | Completely able |
| 3.Can home environment and care strategies support older persons in improving their life skills, including movement, washing, bathing, such as toilets, diet, etc.? | Not at all | Not very competent | General | Almost | Completely able |
| 4.Can the home environment support the elderly in social activities in public spaces? | Not at all | Not very competent | General | Almost | Completely able |
| 5.Can home environment provide private space such as rest for the elderly? | Not at all | Not very competent | General | Almost | Completely able |
| 6.Can the home environment support older persons to exercise their personal preferences, choices and decide when to do something? | Not at all | Not very competent | General | Almost | Completely able |
| 7.Whether light, color, sound and other environmental stimuli are adapted to the elderly to avoid the pressure of the elderly due to environmental stimuli? | Not at all | Not very competent | General | Almost | Completely able |
| 8.Whether the current living environment can be associated with the old people's previous living environment, and feel the past and present their own contact? | Not at all | Not very competent | General | Almost | Completely able |

**D Basic situation of main caregivers**

**D1 Your relationship with the elderly:**

1. Spouse 2. Son 3. Daughter-in-law 4. Daughter 5. Son-in-law

6. Brothers and sisters 7. Others

**D2 Your Gender [Investigator observation and filling]:**

1. Men 2. Women

**D3 What is your age? Years old (based on the past birthday)**

**D4 Your educational level:**

1. No schooling 2. Primary school 3. Junior high school

4. High school/technical secondary school/vocational school 5. College degree

6. Bachelor degree and above

**D5 Do you understand the knowledge and skills of dementia care:**

1. I don't know at all 2. I don't know very well 3. Generally 4. I know better 5. I know very well

**D6 burden of care for the elderly: (Zarit Nursing Burden Scale)**

| **In the following questions, please √ on the most appropriate code you think** | **Never** | **Very few** | **Sometimes** | **Frequently** | **Always** |
| --- | --- | --- | --- | --- | --- |
| 1.Do you think that the patients you care for will ask for too much care? | 1 | 2 | 3 | 4 | 5 |
| 2.Do you think that nursing patients will make their time insufficient? | 1 | 2 | 3 | 4 | 5 |
| 3.Do you think there is pressure between taking care of patients and working hard at home? | 1 | 2 | 3 | 4 | 5 |
| 4.Do you think it is difficult because of the patient’s behavior? | 1 | 2 | 3 | 4 | 5 |
| 5.Do you think that patients are troubled with you? | 1 | 2 | 3 | 4 | 5 |
| 6.Do you think that your patient has affected your relationship with your family and friends? | 1 | 2 | 3 | 4 | 5 |
| 7.Do you worry about the future of patients? | 1 | 2 | 3 | 4 | 5 |
| 8.Do you think that patients depend on you? | 1 | 2 | 3 | 4 | 5 |
| 9.Do you feel nervous when the patient is around you? | 1 | 2 | 3 | 4 | 5 |
| 10.Do you think that your health is affected by nursing patients? | 1 | 2 | 3 | 4 | 5 |
| 11.Do you think that because of nursing patients, you have no time to do your own private affairs? | 1 | 2 | 3 | 4 | 5 |
| 12.Do you think your social interaction is affected by caring for patients? | 1 | 2 | 3 | 4 | 5 |
| 13.Do you give up the idea of inviting friends to come home because the patient is at home? | 1 | 2 | 3 | 4 | 5 |
| 14.Do you think that the patient only expects your care, and you seem to be the only person he / she can rely on? | 1 | 2 | 3 | 4 | 5 |
| 15.Do you think you have no money to care for patients except for your expenses? | 1 | 2 | 3 | 4 | 5 |
| 16.Do you think that you may spend more time nursing patients? | 1 | 2 | 3 | 4 | 5 |
| 17.Do you think it is impossible to live in accordance with your will since the beginning of care? | 1 | 2 | 3 | 4 | 5 |
| 18.Do you wish to leave the patient to others for care? | 1 | 2 | 3 | 4 | 5 |
| 19.Do you have any idea what is good for patients? | 1 | 2 | 3 | 4 | 5 |
| 20.Do you think more should be done for patients? | 1 | 2 | 3 | 4 | 5 |
| 21.Do you think you can do better in caring for patients? | 1 | 2 | 3 | 4 | 5 |
| 22.Overall, how do you evaluate your burden on care? | None | light | Middle | heavy | Extremely heavy |
